# Course of seasonal influenza A/Brisbane/59/07 H1N1 infection in the ferret

**DOI:** 10.1186/1743-422X-7-149

**Published:** 2010-07-09

**Authors:** Alexis McBrayer, Jeremy V Camp, Ron Tapp, Vladimir Yamshchikov, Sheila Grimes, Diana L Noah, Colleen B Jonsson, Carl E Bruder

**Affiliations:** 1 Southern Research Institute, 2000 9th Ave South, Birmingham, AL 35205, USA; 2 Center for Predictive Medicine For Biodefense and Emerging Infectious Disease, University of Louisville, KY 40292, USA; 3 Department of Microbiology, Tumor and Cell Biology, Karolinska Institutet, Nobels väg 16, SE-171 77 Stockholm, Sweden

## Abstract

Every year, influenza viruses infect approximately 5-20% of the population in the United States leading to over 200,000 hospitalizations and 36,000 deaths from flu-related complications. In this study, we characterized the immune and pathological progression of a seasonal strain of H1N1 influenza virus, A/Brisbane/59/2007 in a ferret model. The immune response of the animals showed a dose-dependent increase with increased virus challenge, as indicated by the presence of virus specific IgG, IgM, and neutralizing antibodies. Animals infected with higher doses of virus also experienced increasing severity of clinical symptoms and fever at 2 days post-infection (DPI). Interestingly, weight loss was more pronounced in animals infected with lower doses of virus compared to those infected with a higher dose; these results were consistent with viral titers of swabs collected from the nares, but not the throat. Analyzed specimens included nasal and throat swabs from 1, 3, 5, and 7 DPI as well as tissue samples from caudal lung and nasal turbinates. Viral titers of the swab samples in all groups were higher on 1 and 3 DPI and returned to baseline levels by 7 DPI. Analysis of nasal turbinates indicated presence of virus at 3 DPI in all infected groups, whereas virus was only detected in the lungs of animals in the two highest dose groups. Histological analysis of the lungs showed a range of pathology, such as chronic inflammation and bronchial epithelial hypertrophy. The results provided here offer important endpoints for preclinical testing of the efficacy of new antiviral compounds and experimental vaccines.

## Findings

Every year, influenza virus infects 5-20% of the US population with numerous deaths attributed to primary influenza infection or secondary bacterial pneumonia [1]. The rapid evolution of new influenza virus strains and drug resistant variants demands constant development of treatments as well as reliable animal models allowing for testing of these remedies [2,3]. Although a number of animal models are used for influenza research, ferrets are ideal because they can be readily infected with human isolates of influenza virus (in contrast to mice) and exhibit symptoms similar to humans, such as fever, coughing, sneezing, runny nose, lethargy [4-10], and make a full recovery in 7-10 days [11,12]. Humans and ferrets also share a similar distribution of α-2,6 and α-2,3 linked sialic acid residues, which serve as the receptor for influenza attachment to airway epithelial cells, enabling influenza to use the same cell entry mechanism [5,13,14]. Furthermore, ferrets are large enough to easily monitor aspects of disease progression and yield enough materials for immunological and virological analysis, [6,15-17]. Prior to clinical trials, safety and efficacy need to be demonstrated in two animal models, one non-rodent, making the ferret ideal.

We examined progression of A/Brisbane/59/2007 in ferrets using a full series of endpoints; clinical symptoms, gross and microscopic pathology, virology, and immunology. A/Brisbane/59/07 was obtained from the Centers for Disease Control and Prevention and propagated for 2 days at 34°C in 10-day embryonated hen's eggs [18]. Castrated and de-scented Fitch ferrets (6-8 months of age, 800-1800 grams; Triple F Farms, Sayre, PA) were assigned to one of 6 treatment groups (Table 1) by a weight-matched computer-generated randomization procedure. Five groups were challenged intranasally with increasing doses of A/Brisbane/59/2007, and controls received PBS. Changes in body temperature, body weight, and onset of clinical symptoms were monitored for 7 days after challenge to measure disease progression and severity. Analyzed specimens included blood sera, and excreta samples from nasal and throat swabs from 1, 3, 5, and 7 DPI and tissues from 3 and 7 DPI. Animal studies were approved by Southern Research Institutional Animal Care and Use Committee and met the recommended animal care guidelines.

**Table 1:** Study design and outline of clinical symptoms

Dose Group	Challenge Material	Infectious dose*	Symptoms
1	PBS	--	None

2	A/Brisbane/59/2007	10^3.8^	Discharge, Nose, Serous Discharge, Nose Purulent Discharge, Eye, Clear

3	A/Brisbane/59/2007	10^4.8^	Discharge, Nose, Serous

4	A/Brisbane/59/2007	10^5.8^	Discharge, Nose, Clear Discharge, Eye, Clear

5	A/Brisbane/59/2007	10^6.8^	Discharge, Nose, Serous Discharge, Eye, Clear Sneezing

6	A/Brisbane/59/2007	10^7.8^	Discharge, Nose, Clear Discharge, Nose, Serous Discharge, Eye, Clear Sneezing

* Infectious dose is measured as 50% egg infectious dose per mL (EID_50_/mL)

**Figure 1:**
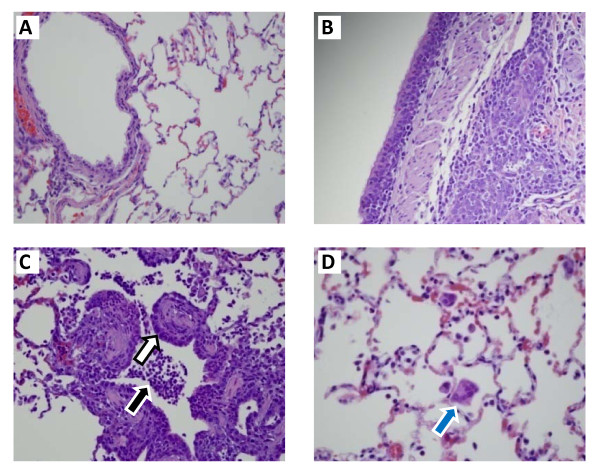
**Clinical Pathology of A/Brisbane/59/2007 infected ferrets**. (A) Control lung tissue; (B) Lung from ferret challenged with 10^3.8 ^EID_50_/ml with chronic inflammation in the bronchial glands; (C). Lung from ferret challenged with 10^4.8 ^EID_50_/ml with bronchiolar epithelial hypertrophy (white arrow) and, neutrophils and macrophages within alveoli and airways (black arrow); (D). Lung of ferret challenged with 10^6.8 ^EID_50_/ml with a syncytium within an alveolus (see blue arrow). Images were taken at 400x magnification

Animals in groups infected with higher doses of influenza experienced greater severity in clinical symptoms compared to those in lower dose groups or control animals (Table 1). Groups infected with influenza demonstrated significant weight loss at 2 through 7 DPI compared to the control group. Animals also exhibited elevated body temperature on 2 DPI. Flu-like symptoms, such as sneezing, and nasal and ocular discharge were seen. Most animals fully recovered by 7 DPI; however, some animals relapsed with a recurrence of clear or serous nasal discharge. Histological analysis of lungs showed a range of pathology, such as bronchiolar epithelial hypertrophy and inflammation. Macroscopic lung lesions consisted of dark/mottled discoloration observed in animals in all dose groups on 3 and 7 DPI. In animals euthanized on 3 and 7 DPI, microscopic lesions consistent with influenza infection were observed in all challenge groups, but not controls. Microscopic lesions in lungs of influenza challenge dose groups consisted of acute inflammation of the alveolus, bronchiole, and bronchiole lumen; chronic inflammation of the alveolus, bronchus, peribronchiolar interstitium and perivascular interstitium; chronic-active inflammation of the alveolus; hemosiderin pigmentation of the perivascular interstitium; type II pneumocyte hyperplasia; bronchiolar hypertrophy; syncytia of the alveolus and bronchiole; and regeneration of the bronchiole. Although the incidence and severity of lesions was variable among dose groups, these parameters tended to be the greatest in animals infected with higher doses of virus. Excluding chronic inflammation of the perivascular interstitium and bronchiolar hypertrophy, which ranged from minimal to mild in severity, lesions noted were minimal in severity (Figure 1).

**Figure 2:**
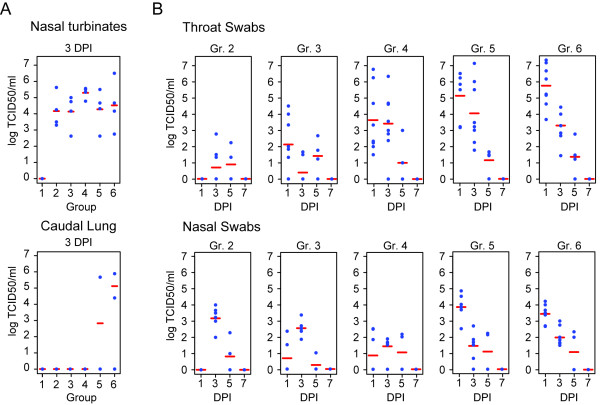
**TCID_50 _Virus Titration Analysis**. Blue dots indicate the titer of individual animals, the red line indicates the average for the animals tested in each group and day. For the caudal lung and nasal turbinates (A), four animals per group were analyzed at 3 DPI. In (B), analysis of throat and nasal swabs isolated at 1, 3, 5, and 7 DPI is shown. Eight samples per group were analyzed on 1 and 3 DPI, and four samples per group were analyzed on days 5 and 7 due to the euthanasia of 50% of the animals on 3 DPI.

Viral load in swabs and tissues was analyzed by titration to determine the TCID_50_. Briefly, MDCK cells (ATCC, clone CCl-34) were grown in DMEM (4.5 g/L glucose, 10% FBS, 1% penicillin/streptomycin, 2 mM L-glutamine, 0.25 M HEPES (all from Gibco)) and seeded at a density of 30,000 cells per well in 96-well plates then incubated at 37°C overnight. For infection, UltraMDCK media (Lonza) (2 μg/mL Trypsin, 1% penicillin/streptomycin, 1% L-glutamine, and 2.5% HEPES) was used. Cells were inoculated with 10-fold serially diluted samples from swabs or tissue homogenates in quadruplicate format. Plates were incubated 3 days at 37°C, 5% CO_2 _and saturated humidity, after which cytopathic effect (CPE) was observed microscopically. The viability was determined using a cell viability assay for the nasal and throat swabs as well as for the nasal turbinates (Cell Titer Aqueous One Reagent, Promega). The lungs were analyzed using A cell based ELISA, since this method proved to be less sensitive to cell toxicity. Briefly, cell plates washed twice with PBS (300 uL/well) and fixed (80% v/v Acetone, 50 μL/well). After three repeats of PBS rinses followed by 10 min RT incubations, mouse-anti-nucleoprotein and mouse anti-matrix protein antibodies (200 ng/mL, 50 μL/well, ATCC) were added. The plates were then incubated for 1 h at RT and washed three times with 300 μL/well of PBS + 0.05% Tween-20 (PBST), after which 200 ng/mL of HRP-conjugated horse anti-mouse IgG (H + L chains) was added to (50 uL per well) and incubated for 1 h at RT. Finally, the plates were developed using TMB 2-component microwell peroxidase substrate kit (KPL). The reaction was stopped using 1 M H_3_PO_4_, and plates were measured at an absorbance of 450 nm. Analysis of nasal turbinates collected on 3 DPI showed similar titers regardless of viral dose administered at challenge. Two animals from group 6 and one animal from group 5 showed presence of virus in the lungs (Figure 2A). Results showed dose-dependent infection in throat swabs for 1 and 3 DPI. Dose dependence is also seen for the nasal swabs on 1 DPI (Spearman-Rho non-parametric testing, *r_s _*> 0.85, Figure 2B and 2C). By 7 DPI, all groups returned to baseline levels, indicating that the animals cleared the infection (Figure 2B and 2C).

**Figure 3:**
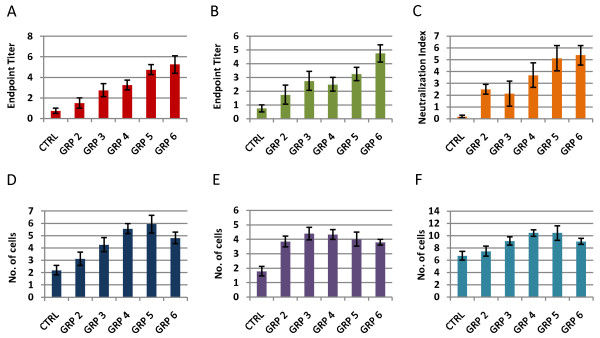
**Humoral and Cellular Immunity**. (A and B) ELISA data show an increase in influenza specific IgM and IgG at 7 DPI compared to mock-infected control animals. These data show that there is a dose-dependent increase in antibody response. Bars indicate the average difference per group between log_2_-transformed end-point dilutions from pre-infection serum and post-infection serum. (C) Neutralization titer analysis was performed in order to detect the presence of influenza-specific neutralizing antibodies in the serum. The presence of neutralizing antibodies was measured only on 7 DPI. No neutralizing antibodies were detected in the sera from control animals. Only 2 out of 4 animals from group 2 and 1 out of 4 animals from group 3 had detectable neutralizing antibodies, while all animals in groups 4, 5, and 6 had detectable neutralizing antibodies. (D, E, and F) The number of neutrophils and lymphocytes, as well as the total number of white blood cells, increased in animals infected with A/Brisbane/59/07 compared to mock infected control animals. Y axis indicates number of cells as 10^3 ^cells per mm^3^.

Immunological parameters were evaluated using virus specific ferret IgG and IgM ELISA on sera collected on 3 and 7 DPI. Briefly, plates were coated with 1:200 dilution of stock virus in PBS overnight at 4°C, blocked with 2% donor goat serum (Sigma Aldrich) in PBS/0.05% v/v Tween-20 for one hour. Ferret serum was then added and 2-fold serially diluted and incubated at 4°C overnight. Anti-ferret IgG or IgM-HRP (1:10,000) (Rockland Immunochemicals) was then added and after a one hour incubation at 37°C, TMB substrate was added, the reaction was stopped using 1 M H_3_PO_4_, and read at absorbance of 450 nm. At 7 DPI, influenza-specific IgM and IgG antibodies increased relative to viral dose administered at challenge (Spearman-Rho non-parametric testing, *r_s _*> 0.94, Figure 3A and 3B). No change between pre-immune and post-immune sera collected at 3 DPI was detected. Neutralization titer analysis was performed to detect influenza-specific neutralizing antibodies in serum. Only sera collected on 7 DPI was evaluated as ELISA results suggested that no neutralizing antibodies were present on 3 DPI. As expected, no neutralizing antibodies were detected in sera from control animals. Only 2 of 4 animals from group 2 and 1 of 4 animals from group 3 had detectable neutralizing antibodies; however neutralizing antibodies were seen in all animals in groups 4, 5 and 6 which were challenged with higher doses of virus (Figure 3C). Hematological analyses were also performed on blood samples collected immediately prior to euthanasia. Results showed an increase in the number of lymphocytes, neutrophils, and the total number of white blood cells in infected groups compared to control (Figure 3D,E,F). There was only a slight increase in the number of basophils and eosinophils in groups 4 and 5 compared to controls.

To conclude, ferrets infected with A/Brisbane/59/2007 H1N1 displayed mild clinical symptoms, with weight loss, sneezing, nasal and ocular discharge as well as histopathological lesions consistent with influenza infection. Histopathology of the lungs indicated a localized immune response. Virus titers exhibit dose dependence, with higher titers early in the course of infection for the higher doses. Lower doses suggest a delay of virus replication in the samples tested. Homogenized nasal turbinates showed a relatively even distribution over time points. In contrast to a recently published study investigating the pathological effects of a single dose of A/Brisbane/59/2007 [19], we detected replicating virus in the lungs, which indicates that this influenza strain is capable of inducing infection in tissues of the lower respiratory tract. High correlation is seen between viral dose at challenge and the immune response detected by virus specific IgG and IgM ELISA, the neutralization index, and to the viral titers of the throat swabs. To conclude, we describe development of a ferret model for analysis of a seasonal influenza strain. The results provide key endpoints for preclinical testing of the efficacy of new antiviral compounds and experiential vaccines.

## Abbreviations

CPE: cytopathic effect; DPI: days post-infection; PBS: phosphate buffered saline; TCID_50_: tissue culture infectious dose 50%.

## Competing interests

The authors declare that they have no competing interests.

## Authors' contributions

AM: immunological analysis, manuscript preparation, JVC: virological and immunological analysis, manuscript preparation, RT: virological and immunological analysis, manuscript preparation, VY: immunological analysis, SG: clinical pathology analysis, DN: virus preparation, CBJ: participated in design of study, review of findings and manuscript preparation, CEB: participated in design, direction of the study, data analysis and manuscript preparation

All authors have read and approved the final manuscript.

## References

[B1] Beigel JH Influenza Crit Care Med 2008 36 2660 2666 10.1097/CCM.0b013e318180b039 18679129 PMC3431208

[B2] Lackenby A Thompson CI Democratis J The potential impact of neuraminidase inhibitor resistant influenza Curr Opin Infect Dis 2008 21 626 638 10.1097/QCO.0b013e3283199797 18978531

[B3] Matsuoka Y Lamirande EW Subbarao K The mouse model for influenza Curr Protoc Microbiol 2009 Chapter 15 Unit 15G 13 10.1002/9780471729259.mc15g03s13 19412911

[B4] Moorman JP Viral characteristics of influenza South Med J 2003 96 758 761 10.1097/01.SMJ.0000084986.13843.52 14515914

[B5] van der Laan JW Herberts C Lambkin-Williams R Boyers A Mann AJ Oxford J Animal models in influenza vaccine testing Expert Rev Vaccines 2008 7 783 793 10.1586/14760584.7.6.783 18665776

[B6] Matsuoka Y Lamirande EW Subbarao K The ferret model for influenza Curr Protoc Microbiol 2009 Chapter 15 Unit 15G 12 10.1002/9780471729259.mc15g02s13 19412910

[B7] Kirkeby S Martel CJ Aasted B Infection with human H1N1 influenza virus affects the expression of sialic acids of metaplastic mucous cells in the ferret airways Virus Res 2009 144 225 232 10.1016/j.virusres.2009.05.004 19447147

[B8] Maher JA DeStefano J The ferret: an animal model to study influenza virus Lab Anim (NY) 2004 33 50 53 10.1038/laban1004-50 15457202

[B9] Reuman PD Keely S Schiff GM Assessment of signs of influenza illness in the ferret model J Virol Methods 1989 24 27 34 10.1016/0166-0934(89)90004-9 2760163

[B10] Yen HL Lipatov AS Ilyushina NA Govorkova EA Franks J Yilmaz N Douglas A Hay A Krauss S Rehg JE Inefficient transmission of H5N1 influenza viruses in a ferret contact model J Virol 2007 81 6890 6898 10.1128/JVI.00170-07 17459930 PMC1933302

[B11] Herlocher ML Truscon R Elias S Yen HL Roberts NA Ohmit SE Monto AS Influenza viruses resistant to the antiviral drug oseltamivir: transmission studies in ferrets J Infect Dis 2004 190 1627 1630 10.1086/424572 15478068

[B12] Svitek N Rudd PA Obojes K Pillet S von Messling V Severe seasonal influenza in ferrets correlates with reduced interferon and increased IL-6 induction Virology 2008 376 53 59 10.1016/j.virol.2008.02.035 18420248

[B13] Leigh MW Connor RJ Kelm S Baum LG Paulson JC Receptor specificity of influenza virus influences severity of illness in ferrets Vaccine 1995 13 1468 1473 10.1016/0264-410X(95)00004-K 8578828

[B14] Nicholls JM Bourne AJ Chen H Guan Y Peiris JS Sialic acid receptor detection in the human respiratory tract: evidence for widespread distribution of potential binding sites for human and avian influenza viruses Respir Res 2007 8 73 10.1186/1465-9921-8-73 17961210 PMC2169242

[B15] Boltz DA Rehg JE McClaren J Webster RG Govorkova EA Oseltamivir prophylactic regimens prevent H5N1 influenza morbidity and mortality in a ferret model J Infect Dis 2008 197 1315 1323 10.1086/586711 18422444

[B16] Lambkin R Oxford JS Bossuyt S Mann A Metcalfe IC Herzog C Viret JF Gluck R Strong local and systemic protective immunity induced in the ferret model by an intranasal virosome-formulated influenza subunit vaccine Vaccine 2004 22 4390 4396 10.1016/j.vaccine.2003.10.054 15474733

[B17] Middleton D Rockman S Pearse M Barr I Lowther S Klippel J Ryan D Brown L Evaluation of vaccines for H5N1 influenza virus in ferrets reveals the potential for protective single-shot immunization J Virol 2009 83 7770 7778 10.1128/JVI.00241-09 19457991 PMC2708649

[B18] Heyward JT Klimas RA Stapp MD Obijeski JF The rapid concentration and purification of influenza virus from allantoic fluid Arch Virol 1977 55 107 119 10.1007/BF01314484 336007

[B19] Rowe T Leon AJ Crevar CJ Carter DM Xu L Ran L Fang Y Cameron CM Cameron MJ Banner D Modeling host responses in ferrets during A/California/07/2009 influenza infection Virology 401 257 265 10.1016/j.virol.2010.02.020 20334888 PMC2862141

